# Psychometric properties of the Arabic version of the perceived prosthodontic treatment need scale: Exploratory and confirmatory factor analyses

**DOI:** 10.1371/journal.pone.0298145

**Published:** 2024-02-06

**Authors:** Rayan Sharka

**Affiliations:** Department of Oral & Maxillofacial Surgery and Diagnostic Sciences, Faculty of Dental Medicine, Umm Al-Qura University, Makkah, Saudi Arabia; Yerevan State Medical University Named after Mkhitar Heratsi, ARMENIA

## Abstract

**Background:**

It is crucial to take into account the concerns of dental patients about their prosthodontic needs when planning the course of treatment. However, there is a dearth of research that examines these needs among adult dental patients.

**Objectives:**

This study aims to translate and validate the perceived prosthodontic treatment need scale (PPTN) in Arabic.

**Methods:**

The 17-item PPTN scale was translated from English into Arabic and completed by 206 patients seeking prosthodontic treatment in a dental hospital in Saudi Arabia. Data collection was carried out in September and October 2023. Exploratory factor analysis (EFA) was used to ascertain the underlying factor structure; a unidimensional scale was hypothesised and tested using confirmatory factor analysis (CFA), including several multiple model fit indices. The assessment of reliability was conducted using Cronbach’s alpha. The convergent and discriminant validity of the final scale were examined.

**Results:**

EFA produced an 11-item scale distributed into three factors that explain 65.43% of the total variance with eigenvalues > 1. All items showed acceptable reliability, ranging from 0.65 to 0.84. The first factor pertained to social issues, while the second item was concerning dental appearance. Lastly, the third factor included functional difficulties associated with tooth loss or dental problems. The results of the CFA demonstrate a satisfactory level of model fit, with the standardised factor loadings ranging from 0.51 to 0.89. Convergent and discriminant validity of the model factors were established.

**Conclusion:**

The translated questionnaire was deemed legitimate and would be useful in comprehending patients’ perceived treatment requirements, hence contributing to the advancement of prosthodontic research and practical implementation.

## Introduction

According to the American Dental Association (ADA), prosthodontics is *“a dental specialty that focuses on the diagnosis*, *treatment*, *planning*, *rehabilitation*, *and maintenance of oral function*, *comfort*, *appearance*, *and health for patients who have clinical conditions related to missing or deficient teeth and/or oral and maxillofacial tissues*. *This specialty utilises biocompatible substitutes to address these conditions*” [1]. Prosthodontic treatments primarily focus on addressing issues related to teeth loss and its consequences, including difficulties with chewing, unsatisfactory aesthetic appearance, and the necessity for replacement with dental prostheses such as implant-supported prostheses, fixed tooth-supported prostheses, and removable dentures [1]. The persistence of the need for prosthodontic treatment is very unlikely to diminish. While the prevalence of edentulism in industrialised nations is expected to decline and be centred among the older population, there is a projected increase in the number of individuals with partial tooth loss, necessitating prosthodontic interventions to maintain their oral health [2–4]. The acquisition of required prosthodontic treatment is a significant determinant in sustaining optimal oral health, hence impacting an individual’s overall satisfaction and quality of life [5–8].

The main goals of prosthodontic treatment are to meet the specific needs of each person. Also, prosthodontic treatments extend beyond addressing functional requirements associated with chewing and mastication. Furthermore, it provides aesthetic enhancement, ultimately influencing social and psychological requirements. Simultaneously, it also has financial ramifications [8]. Multiple studies in Saudi Arabia (SA) have examined the prevalence of complete tooth loss (edentulism) and partial tooth loss among the adult population. This research indicates a consistent need for prosthodontic treatment [9–11]. As an example, Almusallam and AlRafee [10] conducted a multicenter study in the capital city of SA, Riyadh. Among 618 subjects, 69% had one or more teeth missing. Moreover, Gad et al. [9] investigated the prevalence of partial edentulism in the eastern region of SA. They found that nearly half of their test subjects (47%) had Kennedy Class I [9].

Moreover, individuals who experience tooth loss and need dental treatment have a diminished quality of life as a result of compromised oral function, heightened levels of discomfort, and increased anxiety [12, 13]. Several recognised scales, such as the Oral Health Impact Profile (OHIP) and the Geriatric Oral Health Assessment (GOHAI), may be used to evaluate dental treatment requirements [14, 15]. The OHIP examines the social impact and psychological effects, while the GOHAI analyses oral health issues. Nevertheless, there is currently no well-recognised tool that explicitly evaluates the need for prosthodontic treatment.

The use of a validated scale pertaining to measuring prosthodontic treatment needs has the potential to enhance comprehension of the anticipated treatment quality from the standpoint of patients. Numerous reports showed that patient-oriented or patient-reported outcomes (PROs) are being used more often by scholars and dental practitioners to comprehensively assess the effects of illnesses and medical treatments on patients [16–18]. This approach complements the traditional disease-oriented outcomes by providing a more holistic understanding of the effect that treatments have on the patient [16]. Therefore, using a structured approach for assessing the difficulties experienced by patients may greatly facilitate the formulation of appropriate and customised dental treatment plans.

While many existing validated instruments have been used to examine the demands of dental patients, there is only one scale specifically designed by prosthodontic academics and specialists perceived prosthodontic treatment needs (PPTN) to assess the treatment needs of patients seeking prosthodontic treatment. At present, the original PPTN scale developed by Soo et al. [19] has been validated in the English language and has been examined among adult patients in Malaysia, and this new scale has not undergone translation or validation processes in any other language or nation [19]. Also, the psychometric properties, including factor structure stability and convergent and discriminant validity of the scale, were not conducted in the previous study [19]. The psychometric properties of a scale refer to its characteristics that indicate its level of validity, reliability, and responsiveness [20]. Moreover, the translation process with cross-cultural adaptation is a crucial measure to guarantee linguistic equivalence and comparability of patients’ requirements across multiple cultures [21]. Therefore, this study aimed to translate the PPTN scale into the Arabic language and conduct a psychometric properties analysis of the translated scale. The hypothesis of the study is that the Arabic-translated PPTN scale will demonstrate adequate factor structure.

## Materials and methods

### Study setting

**Fig 1:**
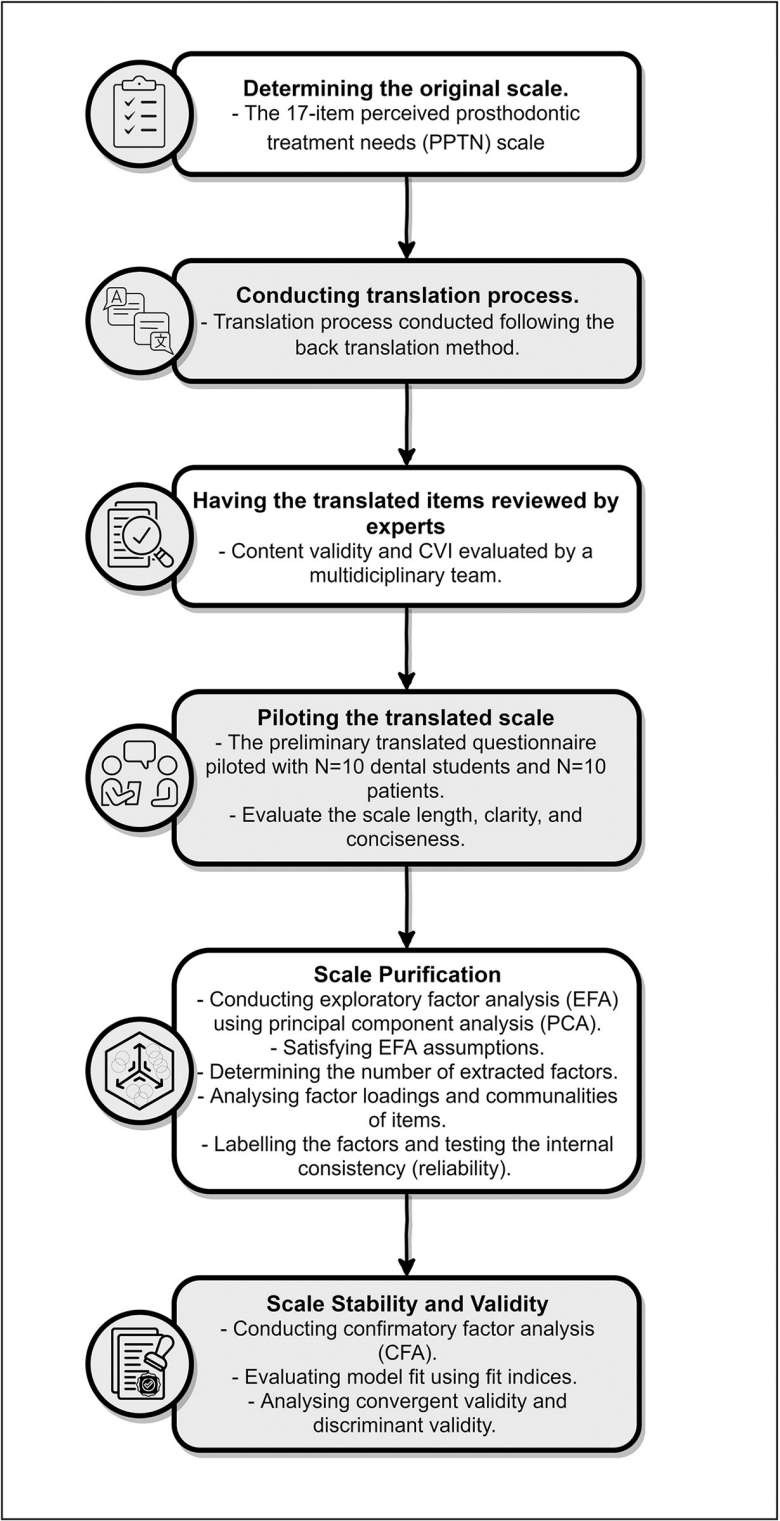
A flow chart of key stages in designing and analysing the study.

The biomedical research ethics committee of Umm Al-Qura University has approved this research project with clearance no. [HAPO-02-K-012-2023-09-1705]. The present research was a cross-sectional study carried out at the Faculty of Dental Medicine and Dental Teaching Hospital of Umm Al-Qura University, located in the western region of Saudi Arabia. Fig 1 illustrates a flow chart outlining the key stages involved in this study to design and translate the questionnaire, as per the recommendations provided in prior studies.

### The original scale

The original 17-item PPTN was used in this study [19]. A group of academic experts and dental specialists with specialised knowledge in prosthodontics, restorative dentistry, and dental public health undertook the development of the original scale [19]. A thorough review of pertinent literature served as the basis for the scale’s construction. Moreover, a series of qualitative interviews were also conducted in order to establish a comprehensive pool of relevant items [19]. The scale has sixteen items designed to assess the factors influencing the decision to pursue prosthodontic therapy, together with an additional item to evaluate the overall need for such treatment.

### Translations and cross-cultural adaptations

The process of translating and cross-culturally adapting the 17-item PPTN followed the back translation methodologies outlined in the previous studies [21, 22]. It has been suggested that the translation process should be iterative and include more than literal translation [21, 22]. It is also important to take cultural variations into account to maintain the content validity of the scale on a conceptual level across various societies. Two faculty members who are experienced in developing questionnaires and proficient in English were requested to independently translate the original scale items from English to Arabic. Two translations were acquired. A tentative Arabic translation was achieved after a consensus meeting between two translators. Subsequently, two different faculty members who are native speakers of English were requested to undertake the task of translating the preliminary translated scale back into its English language [23]. No major differences were identified between the two versions, and after a series of revisions, the final version was produced with a few modifications. Occasionally, the process of cross-cultural adaptation necessitates the exclusion, addition, or alteration of descriptors [22]. Adopting the current PPTN scale for cross-cultural use in the Arabic context is a suitable approach to creating a reliable evaluation scale. This scale allows dental practitioners to evaluate each patient within the patient’s own cultural context. As an example, in item 3, the term “partner” was substituted with “wife/husband” to account for cultural variations. Furthermore, the term “unhappy” was omitted in item 5 because in Arabic, the terms “unhappy” and “sad” are identical [see S1 File].

### Item relevance and content validity

A multidisciplinary team from the author’s dental school, consisting of six faculty members in oral surgery, prosthodontics, public health, and endodontics whose first language was Arabic and fluent in English, was asked to review the translated scale for relevance and content validity following the content validity process proposed by the previous study [24]. The team conducted an independent analysis to assess the appropriateness of the scale within the Arabic context. Additionally, they sought a critical evaluation of the factor and its constituent items. The factor characteristics were obtained from the original scale. The relevance rating of each item is transformed into a 4-point scale, where a rating of 1 indicates that the item is not relevant to the measured factor and a rating of 4 indicates that the item is highly relevant to the measured factor. Only the items that met the criterion of having a content validity index (I-CVI) value greater than or equal to 0.8 were included in the analysis [24, 25]. All items fulfilled this criterion and attained an acceptable level of content validity. The relevance ratings assigned to an item scale by six experts can be found in [see S2 File].

Subsequently, a pilot of the Arabic version of the scale was conducted with a cohort of 10 dental trainees (interns) who had no prior familiarity with the measure on September 5, 2023. The questionnaire was piloted with dental interns to help in scrutinising the phrasing of questions and identify any items that may provide possible difficulties for the reader. The dental interns demonstrated a comprehensive understanding of the meaning of all items, exhibiting no concern. Also, a total of 10 participants randomly chosen from the patient waiting area were asked to complete a paper questionnaire in the presence of researchers. Generally, patients believed that the questionnaire items were worded in a straightforward manner and were easily comprehensible.

### Administering the final scale to participants

Adult patients who attended general dentistry clinics and prosthodontic specialty clinics to seek or receive prosthodontic treatment at the time of questionnaire administration between September 6 and October 8, 2023, were asked to participate using convenience sampling. Exclusion criteria included patients below the age of 18 who were unable to offer informed consent. Also, patients who lacked proficiency in reading and understanding Arabic.

The determination of the sample size was performed based on prior research and established statistical guidelines [26–29]. These suggestions proposed that it is preferable to have a respondent-to-item ratio of 10:1. The ratio used in this study led to a sample size of 170 patients. To account for any missing data and to achieve a response rate that aligns with the desired sample size, the sample size was increased to N = 210.

The final questionnaire had the following sections: The first section included participants’ information sheet and consent form statements. Section two consists of a set of three demographic questions pertaining to the participant’s gender and age, using a categorical measurement scale. The third section of the questionnaire included the use of 17 psychometric items to assess the perceived prosthodontic treatment need. The questionnaire employed a five-point Likert scale; the first 16 items have response options ranging from "never" to "very often", similar to the original PPTN scale [19]. The last item, which measures the overall patients’ perceptions of prosthodontics treatment need, also has a five-point Likert scale ranging from “none” to “very high need.” The full questionnaire can be found in [see S2 File].

### Statistical analysis

The statistical analysis was conducted using the SPSS programme (Version 27; IBM Corp., Chicago, IL, USA). Descriptive statistics were computed to analyse categorical variables, such as gender and age. These statistics included frequency distributions and percentages. Because this new scale was tested in Arabic for first time, the initial psychometric analysis was computed using exploratory factor analysis (EFA) by applying principal component analysis (PCA) followed by a varimax (orthogonal) rotation method to determine the factor structure and parsimonious factorability of this scale [27, 29, 30]. PCA is a multivariate statistical method used to identify the underlying structure and transform high-dimensional data into a more manageable and lower-dimensional representation while preserving as much information as possible by leveraging the relationships between variables [27, 29, 30]. The varimax rotation was used to achieve an optimum and simplified structure, ensuring that each component defines a discrete cluster of correlated variables, hence facilitating simpler interpretation [27]. To satisfy EFA assumptions, the sampling adequacy measure (MSA) was examined first. A threshold of 0.80 or higher is considered adequate [26]. The second test conducted was the Bartlett test for sphericity. A significant value (P < 0.05) determines the presence of correlations among variables that meet the PCA assumption [26]. The determination of the number of extracted components to retain was guided by many criteria, including eigenvalues greater than 1, prior empirical research, the proportion of variance explained over 60%, and the scree plot analysis [26, 29]. Items that had loading values over 0.4 were considered to have practical significance and were preserved [26]. The analysis removed any item that had loading values over 0.35 on multiple factors or falling below 0.4 on a single factor [26, 29]. In addition, each item should have communalities greater than 0.50 for retention in the analysis [26]. Cronbach’s alpha (α) coefficient was calculated to examine the reliability of the extracted factors. A threshold above 0.6 should obtained for each factor [26].

Confirmatory factor analysis (CFA) was estimated to validate the extracted factors and to further assess the psychometric properties of the scale using the Amos software (Version 26; IBM Corp., Chicago, IL, USA). The adequacy of the model fit was evaluated by five common model fit indicators, including χ2 /df ≤ 3.0, the Root Mean Square Error of Approximation (RMSEA), Comparative Fit Index (CFI), Bentler–Bonett normed fit index (NFI), Tucker-Lewis Index (TLI), and Standardized Root Mean Square Residual (SRMR) [27, 30, 31]. A satisfactory model fit was indicated by values of RMSEA below 0.08, as well as CFI and TLI over 0.90. An SRMR score of 0.07 is considered acceptable [28, 31–33]. These fit indices provide an estimate of the quality of fit or the extent to which the model deviates from an ideal fit model [28, 31, 32].

Convergent validity of the scale is established if the following conditions are met: First, the average variance extracted (AVE) should be greater than 0.50 for each factor. Second, the path loadings for each item of each factor in the model must be greater than 0.5. Lastly, composite reliability (CR) must be at least 0.60 [34]. Discriminant validity is established if the square root of (AVE) for each factor is greater than the inter-factor correlation [34]. The single item measuring the overall patients’ perceptions of prosthodontic treatment need was not included in the EFA and CFA analyses, as it is advisable to have at least 3 items per factor [28, 31–33].

## Results

### Participants demographics

A total of 220 questionnaires was administered to patients and 206 patients completed the surveys, and their responses were used for data analysis. This resulted in an overall response rate of 93.6%. Out of the 206, 110 (53.4%) of the participants were female, and the remaining 96 (46.6%) were male. About one-third of participants, 66 (32%), were in the age range of 25–34 years old, while 45 (27%) were above 45 years old.

### Exploratory factor analysis (EFA)

The coefficient for the MSA was determined to be 0.818, suggesting that the sample size was adequate. The Bartlett’s test for sphericity yielded statistically significant results (*X*^2^ (55) = 887.526, p < 0.001), indicating that the assumptions for EFA were satisfied.

**Fig 2:**
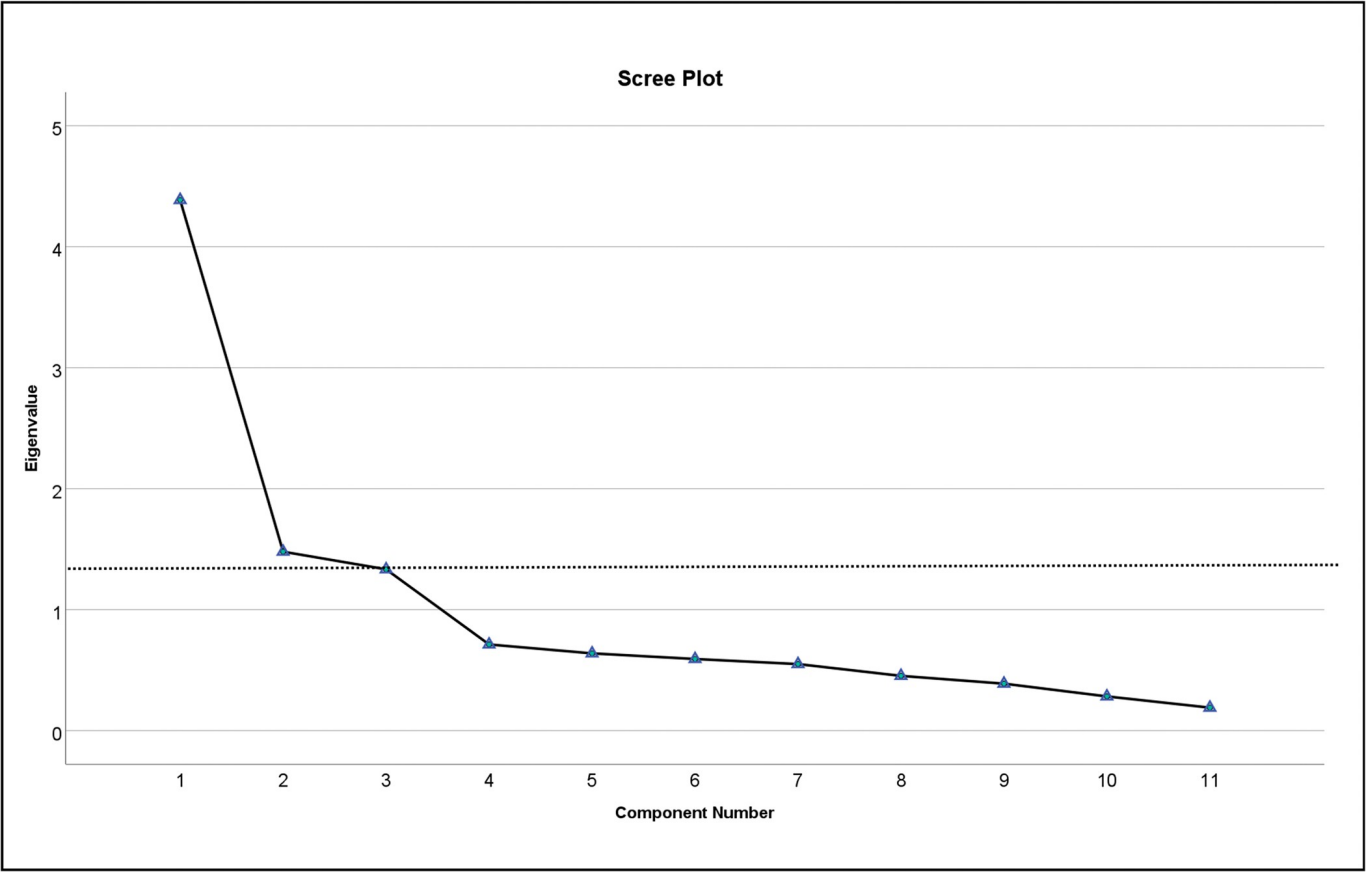
Scree plot of the (EFA) representing the items with eigenvalues plotted. The inspection of the scree plot shows that three extracted factors are acceptable, as the "elbow" of the line found after three factors.

The initial EFA round conducted on the 16 items revealed that five items (Q4, Q5, Q6, Q7, and Q9) had factor loadings > 35 on more than one factor. Consequently, these items were excluded from further consideration in the analysis. The final EFA yielded three factors, including a total of 11 items, each factor with characteristic eigenvalues values greater than one. These factors together account for 65.43% of the overall variance. The three factors extracted in this study were further validated using the scree plot analysis, as seen in (Fig 2), and corroborated by the results obtained from the original English version of the scale. Table 1 displays the rotated (orthogonal) component matrix along with significant loading and communality (*h*^2^) for each item on the three factors extracted.

**Table 1:** Results of EFA and communalities.

Factors	Loading			*h*^*2*^ ^a^
Factor 1. Social issues				
Item 1. Avoid going out	.874			.781
Item 2. Social and leisure issue	.848			.785
Item 3. Concerned with what others perceive	.660			.605
Factor 2. Appearance concerns				
Item 8. Disliked teeth show		.734		.655
Item 10. Spent time observing teeth		.587		.464
Item 11. Disliked teeth colour		.763		.610
Item 12. Disliked smile		.853		.788
Item 13. Not attractive teeth		.784		.684
Factor 3. Functional problems				
Item 14. Chewing problem.			.745	.610
Item 15. Avoid certain types of food.			.788	.648
Item 16. Discomfort due to food sticking			.729	.567
% of variance	27.62	21.09	16.72	

Extraction Method: Principal Component Analysis, Rotation Method: Varimax with Kaiser Normalization. Rotation converged in 5 iterations

^a^ *h*^*2*^: Communality, blank cell: Items with loadings <0.35.

### Factors reliability

The Cronbach’s alpha score for the three factors was excellent. Factor 1 was α = 0.807; the second factor was 0.845; and the third factor was 0.659.

### Factors labelling

Upon careful examination of all three items grouped together in factor one, the following description may be formulated: Prosthodontic treatment is essential for individuals to actively participate in social activities and foster interpersonal relationships. This factor is labelled as social issues. The second factor was indicated by a set of five items. These items pertain to the individual’s subjective need for prosthodontic treatment, which arises from concerns over the smile, colour of teeth, and overall dental appearance. This factor was labelled as appearance concerns. The third factor consisted of three items related to the necessity of prosthodontic treatment to restore mastication and be able to eat preferred foods. This factor is referred to as functional problems.

### Confirmatory factor analysis (CFA)

The estimated model had a satisfactory level of fit and yielded a statistically significant outcome for the Chi-square test (χ2 = 93.651, df = 41, p < .0001). The ratio of χ2/df was 2.284, indicating a satisfactory model fit. The model fit indices were as follows: CFI = 0.938, NFI = 0.897, TLI = 0.917, RMSEA = 0.079, and SRMR = 0.060. Based on the CFA estimate, it can be determined that the measurement model exhibits a satisfactory level of fit.

### Convergent and discriminant validity

The AVE values for the three factors were above 0.5, indicating a satisfactory level of convergent validity (Table 2). Additionally, the composite reliability (CR) values surpassed 0.6, indicating a high level of internal consistency and reliability in the measurement model. The model fulfils all the criteria for convergent validity (Table 2).

**Table 2:** Summarised results of descriptive measures of factors and convergent validity.

Factors	Number of items	Cronbach alpha (α)	(CR)^a^	AVE^b^	Mean
Social issues	3	.807	.821	.607	2.15
Appearance concerns	5	.845	.847	.533	2.60
Functional problems	3	.659	.665	.502	3.36

^a^ CR: composite reliability

^b^ AVE: average variance extracted

**Fig 3:**
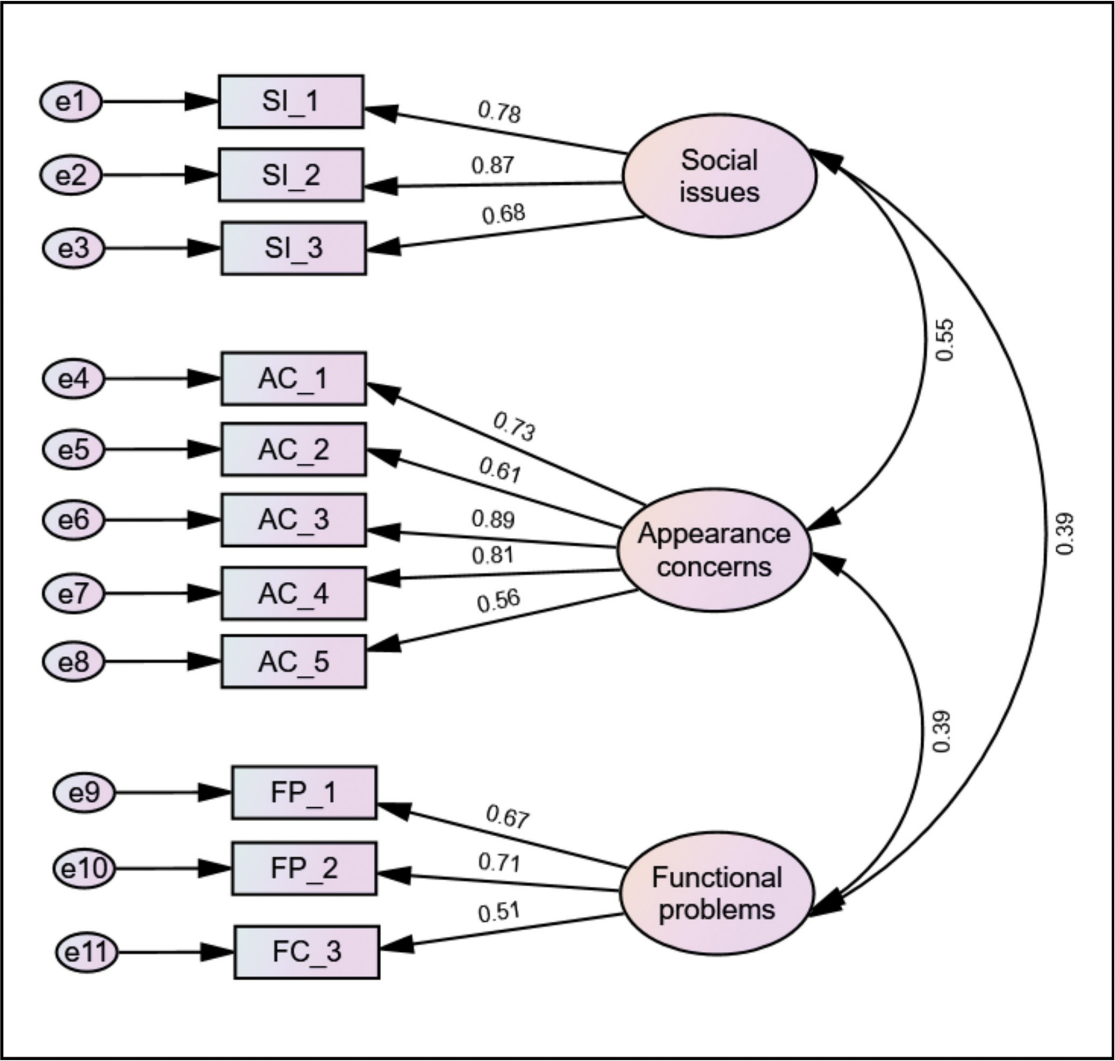
Standardised estimates of the CFA.

Similarly, the standardised path loadings for each item were found to be larger than 0.5, suggesting a strong relationship between the observed variables and their underlying factor (Fig 3).

The discriminant validity of the scale was achieved in this study. The square root values of AVE for the three factors were greater than the correlation estimate between factors (Table 3).

**Table 3:** Correlation matrix of factors.

Factors	Social issues	Appearance concerns	Functional problems
Social issues	**0.779** *	.552	.392
Appearance concerns	.552	**0.730** *	.386
Functional problems	.392	.386	**.635** *

*Diagonal values are the square roots of AVE while non-diagonal values are correlation estimates between factors.

## Discussion

The main objective of this study was to conduct the translation and evaluation of the psychometric properties of the Arabic version of the PPTN scale. Conducting such investigations is essential due to the inherent variety across cultures [20]. Individuals exhibit unique expressions and reactions in response to various stimuli [21]. The significance of adjusting the scale to the treatments and prevailing culture of a specific nation is evident. Furthermore, the use of incompletely translated scales or unverified translations might lead to misinterpretations, thereby compromising the well-being of patients [20, 21].

In this study, the PPTN-Arabic scale was validated and allocated to three distinct factors. The identification of differences between the Arabic-translated version of PPTN and the original PPTN scale proved to be intriguing [19]. The main difference was identified in factor 1 (social concerns). This factor had a substantial correlation with just three items pertaining to social concerns, i.e., avoid going out, problems in joining social events, and concerns about others’ perceptions. Surprisingly, in contrast with the original scale, items related to psychological issues such as depression, embarrassment, lack of confidence, and comparing teeth with others failed to correlate with this factor. This implies that the implementation of prosthodontic treatment among this sample significantly influenced their social well-being, facilitating their active participation in social interactions and enabling them to reintegrate into their normal daily activities. Previous studies have confirmed this factor by finding that social isolation and loneliness have been linked with fewer teeth [35]. According to a systematic review of patients’ perspectives on tooth loss, edentulism and oral rehabilitation, it was shown that prosthodontic treatment has the capacity to restore patients to a state of normality in their social lives [36]. Similarly, a recent empirical study found that the use of dental prostheses has the potential to mitigate the likelihood of experiencing social isolation, particularly among those who have had extensive tooth loss [37]. Thus, prosthodontic treatment leads to an improvement in their overall quality of life and facilitates their engagement in conversations with others, alleviating the persistent anxiety associated with disclosure.

Nevertheless, the phenomenon of psychological problems disappearing is noteworthy and warrants more exploration in future studies. There are some potential explanations for this result. It may be inferred that the participants in this research did not prioritise the psychological aspect while seeking prosthodontic treatment due to their age, as approximately half of the participants were > 35 years old. The existing body of literature has firmly shown that young and adolescents’ individuals were more susceptible to psychological issues such as depression and anxiety [38]. Another possible justification is that the position of missing teeth may have a greater influence on psychological factors compared with other factors. A previous study conducted among adults’ patients seeking dental treatment revealed that the location of missing teeth, especially the anterior teeth, has more negative consequences for psychosocial problems [12].

The second factor identified in this study included five items pertaining to dental aesthetics, specifically focusing on smile problems, disliked colours of teeth, and perceived unattractiveness of teeth. Prior studies have shown that missing teeth have a detrimental impact on an individual’s overall quality of life [39, 40]. In light of the current social milieu, it is evident that individuals have an increased propensity to adhere to prevailing norms regarding modes of self-expression. Consequently, the significance of dental aesthetics in attaining acceptance from society has become more paramount. However, Besfor & Sutton (2018) categorise patients into two groups: “*idealists*” and “*realists*” [41]. It is important for dental professionals to recognise the preferences of patients who place a high value on natural aesthetics while simultaneously dismissing the influence of social media influencers and deceptive advertisements that promote idealised Hollywood smiles, which could have irreversible detrimental effects on oral health and teeth [29, 41, 42]. Furthermore, sociodemographic and cultural variables may influence the need for dental aesthetics [43]. It is crucial to conduct future cross-national research including various age groups, especially younger generations, in order to acquire a comprehensive knowledge of views towards dental aesthetics.

The third factor included three items related to difficulties in chewing, avoidance of certain types of food and food getting stuck between teeth. Identical items were grouped on the original scale. In a broad sense, the loss of teeth has adverse effects on vital oral functions and leads to a decline in chewing efficiency [8]. Data from previous empirical studies showed that malnourished individuals have a substantial decline in the number of teeth [44, 45]. One of the primary benefits of prosthodontic treatment is its notable impact on the restoration of mastication, eating enjoyment, and phonetic capabilities [46, 47]. This factor was consistent with a cross-sectional study that examined the knowledge and attitudes of individuals towards prosthodontic treatment in the western region of Saudi Arabia [48]. The study revealed that a significant proportion of participants expressed an intention to have prosthodontic procedures for the purposes of improving chewing, followed by enhancing dental appearance [48].

Furthermore, this finding has important implications for the implementation of dental procedures. Dental practitioners should educate patients about the detrimental effects of tooth loss on their remaining teeth and oral functions. Prior research revealed a lack of understanding among partly edentulous patients of the negative outcomes associated with tooth loss, highlighting the need for public education on this matter [49].

With regard to the reliability of the extracted factors, in this study, Cronbach’s alpha values ranged from 0.659 to 0.845, which supported the reliability of the factors [50]. This result was comparable with the original PPTN scale and previous studies [19, 29]. In addition, this research provided additional confirmation of the three-factor model that emerged from the EFA. The CFA results confirmed the number of fundamental factors inside the PPTN scale and the structure of the item-factor relationships [32]. Also, the convergent and discriminant validity of the PPTN factors were also confirmed. Thus, this scale demonstrated sufficient validity.

Moreover, the PPTN scale was first created, validated in English, and tested among a sample of Malaysian adults [19]. The current research extends its scope beyond simple translation to include an examination of this crucial scale in a different cultural context. Additionally, the study provided empirical support for the satisfactory psychometric properties of the Arabic version of the scale. The validation of a translated scale serves an imperative role in verifying that the outcomes of cross-cultural empirical investigations are not attributed to translation faults but rather to cultural variations or commonalities [21]. The methodologies used in this research, including back translation, a pilot study, and a factor analysis study, were found to be endorsed by the World Health Organisation (WHO) and international experts in many studies conducted across various cultural contexts [21, 51].

The research confirmed three factors that were specifically designed for patients seeking prosthodontics treatment. Hence, it is important to be careful when applying this scale to other dental specialties. Furthermore, the study was carried out among sample from a single dental hospital located in the western part of Saudi Arabia. To improve the generalisability of the scale, it is recommended that further research be undertaken in different regions and several dental hospitals across other Arabic counties. Also, although the sample size used in this study was deemed sufficient for conducting (EFA) and (CFA) based on the recommended guidelines, it is advisable for future research to employ a higher sample size, particularly for CFA, due to the sensitivity of certain fit indices to sample size. Finally, items related to psychological concerns neither correlate with social concerns factor nor are identified as distinct factor. The phrases that refer to this particular issue need to be revised and retested in future research endeavours.

## Conclusions

The presence of a validated scale to measure the reason for seeking prosthodontic treatment has several implications in clinical settings as an essential determinant for optimising efficiency, minimising resource allocation, and avoiding superfluous interventions. Moreover, it will help dentists prioritise the fulfilment of patients’ expectations and select the most suitable treatment modalities.

In light of identifying the most salient factor, it is crucial for dentists to prioritise functional aspects and guarantee that dental prosthetics adequately reinstate patients’ oral functioning.

The validated tool may enhance the understanding of the perceived prosthodontic requirements of patients prior to the initiation of any dental intervention.

## Supporting information

S1 File The Arabic translation version of PPTN scale. (PDF)

S2 File The relevance ratings on the item scale by six experts. (PDF)

## References

[1] Taylor T, Bergen S, Conrad H, Goodacre C, Piermatti J. What is a Prosthodontist and the Dental Specialty of Prosthodontics? 2018. Available from: https://www.prosthodontics.org/assets/1/7/1.What_is_a_Prosthodontist_and_the_Dental_Specialty_of_Prosthodontics__-_approved1.pdf.

[2] Douglass CW, Shih A, Ostry L. Will there be a need for complete dentures in the United States in 2020? J Prosthet Dent. 2002;87:5–8. doi: 10.1067/mpr.2002.121203 11807476

[3] Basker RM, Davenport JC, Thomason JM. Prosthetic Treatment of the Edentulous Patient. 5th ed. West Sussex: John Wiley & Sons; 2011.

[4] Sharka RM, Alsaggaf AU. A Cross-sectional Study of Self-confidence Differences in Removable Prosthodontic Treatment among Undergraduate Students of a Saudi Dental School. Open Dent J. 2023;17:e18742106277123. doi: 10.2174/0118742106277123231123065534

[5] Myint Oo KZ, Fueki K, Yoshida-Kohno E, Hayashi Y, Inamochi Y, Wakabayashi N. Minimal clinically important differences of oral health-related quality of life after removable partial denture treatments. J Dent. 2020;92:103246. doi: 10.1016/j.jdent.2019.103246 31738967

[6] Ali Z, Baker S, Barabari P, Martin N. Efficacy of Removable Partial Denture Treatment: A Retrospective Oral Health-Related Quality of Life Evaluation. Eur J Prosthodont Restor Dent. 2017;25:101–7. doi: 10.1922/EJPRD_01669Ali07 28590096

[7] Swelem AA, Abdelnabi MH. Attachment-retained removable prostheses: Patient satisfaction and quality of life assessment. J Prosthet Dent. 2021;125:636–44. doi: 10.1016/j.prosdent.2020.07.006 32893014

[8] Sharka R, Abed H, Hector M. Oral health-related quality of life and satisfaction of edentulous patients using conventional complete dentures and implant-retained overdentures: An umbrella systematic review. Gerodontology. 2019;36:195–204. doi: 10.1111/ger.12399 30875108

[9] Gad MM, Abualsaud R, Al-Thobity AM, Al-Abidi KS, Khan SQ, Abdel-Halim MS, et al. Prevalence of partial edentulism and RPD design in patients treated at College of Dentistry, Imam Abdulrahman Bin Faisal University, Saudi Arabia. Saudi Dent J. 2020;32(2):74–9. doi: 10.1016/j.sdentj.2019.07.002 32071535 PMC7016229

[10] Almusallam SM, AlRafee MA. The prevalence of partial edentulism and complete edentulism among adults and above population of Riyadh city in Saudi Arabia. J Fam Med Prim Care. 2020;9:1868–72. doi: 10.4103/jfmpc.jfmpc_1209_19 32670932 PMC7346903

[11] AlZarea BK. Dental prosthetic status and prosthetic needs of geriatric patients attending the College of Dentistry, Al Jouf University, Kingdom of Saudi Arabia. Eur J Dent. 2017;11:526–30. doi: 10.4103/ejd.ejd_69_17 29279682 PMC5727741

[12] Imam AY. Impact of Tooth Loss Position on Oral Health-Related Quality of Life in Adults Treated in the Community. J Pharm Bioallied Sci. 2021;13:969–74. doi: 10.4103/jpbs.jpbs_87_21 35017909 PMC8686957

[13] Anbarserri NM, Ismail KM, Anbarserri H, Alanazi D, AlSaffan AD, Baseer MA, et al. Impact of severity of tooth loss on oral-health-related quality of life among dental patients. J Fam Med Prim Care. 2020;9:187–91. doi: 10.4103/jfmpc.jfmpc_909_19 32110588 PMC7014884

[14] Slade GD, Spencer AJ. Development and evaluation of the Oral Health Impact Profile. Community Dent Health. 1994;11:3–11. https://pubmed.ncbi.nlm.nih.gov/8193981/ 8193981

[15] Atchison K, Dolan T. Development of the Geriatric Oral Health Assessment Index. J Dent Educ. 1990;54:680–687. https://pubmed.ncbi.nlm.nih.gov/2229624/ 2229624

[16] Mittal H, John MT, Sekulić S, Theis-Mahon N, Rener-Sitar K. Patient-Reported Outcome Measures for Adult Dental Patients: A Systematic Review. J Evid-Based Dent Pract. 2019;19:53. doi: 10.1016/j.jebdp.2018.10.005 30926102 PMC6442935

[17] Palaiologou A, Kotsakis GA. Dentist-Patient Communication of Treatment Outcomes in Periodontal Practice: A Need for Dental Patient–Reported Outcomes. J Evid Based Dent Pract. 2020;20:101443. doi: 10.1016/j.jebdp.2020.101443 32473794

[18] De Bruyn H, Raes S, Matthys C, Cosyn J. The current use of patient-centered/reported outcomes in implant dentistry: a systematic review. Clin Oral Implants Res. 2015;26:45–56. doi: 10.1111/clr.12634 26385620

[19] Soo SY, Lee SM, Tew IM, Mohd Dom Tuti Ningseh, Yahya NA. Development and validation of a questionnaire on perceived prosthodontic treatment needs in Malaysian adults. J Prosthet Dent. 2023. doi: 10.1016/j.prosdent.2023.06.003 37468369

[20] DeVellis RF. Scale Development: Theory and Applications. 4^th^ ed. Thousand Oaks: SAGE Publications; 2017.

[21] Popovac A, Pficer JK, Stančić I, Vuković A, Marchini L, Kossioni A. Translation and preliminary validation of the Serbian version of an ageism scale for dental students (ASDS-Serb). Spec Care Dentist. 2022;42:160–9. doi: 10.1111/scd.12656 34582583

[22] Piaton S, Barlow P, Kossioni A, Tubert-Jeannin S, Marchini L. Translation and preliminary validation of a French version of an ageism scale for dental students. Gerodontology. 2022;39:1–6. doi: 10.1111/ger.12606 34275154

[23] Acquadro C, Conway K, Hareendran A, Aaronson N. Literature Review of Methods to Translate Health-Related Quality of Life Questionnaires for Use in Multinational Clinical Trials. Value Health. 2008;11:509–21. doi: 10.1111/j.1524-4733.2007.00292.x 18179659

[24] Yusoff MSB. ABC of Content Validation and Content Validity Index Calculation. Educ Med J. 2019;11:49–54. doi: 10.21315/eimj2019.11.2.6

[25] Polit DF, Beck CT. The content validity index: Are you sure you know what’s being reported? critique and recommendations. Res Nurs Health. 2006;29:489–97. doi: 10.1002/nur.20147 16977646

[26] Hair J, Black W, Anderson R, Babin B. Multivariate Data Analysis. 7th ed. Edinburgh: Pearson Education Limited; 2014.

[27] Yong AG, Pearce S. A Beginner’s Guide to Factor Analysis: Focusing on Exploratory Factor Analysis. Tutor Quant Methods Psychol. 2013;9:79–94. doi: 10.20982/tqmp.09.2.p079

[28] Sarmento RP, Costa V. Confirmatory factor analysis-a case study. arXiv. 2019;1905.05598. doi: 10.48550/arXiv.1905.05598

[29] Sharka R, San Diego J, Nasseripour M, Banerjee A. Factor analysis of risk perceptions of using digital and social media in dental education and profession. J Dent Educ. 2023;87:118–29. doi: 10.1002/jdd.13085 36036230

[30] Watkins MW. Exploratory Factor Analysis: A Guide to Best Practice. J Black Psychol. 2018;44:219–46. https://psycnet.apa.org/doi/ doi: 10.1177/0095798418771807

[31] Boateng GO, Neilands TB, Frongillo EA, Melgar-Quiñonez HR, Young SL. Best Practices for Developing and Validating Scales for Health, Social, and Behavioral Research: A Primer. Front Public Health. 2018;6:149. doi: 10.3389/fpubh.2018.00149 29942800 PMC6004510

[32] Mvududu NH, Sink CA. Factor Analysis in Counseling Research and Practice. Couns Outcome Res Eval. 2013;4:75–98. doi: 10.1177/2150137813494766

[33] Shek DTL, Yu L. Confirmatory factor analysis using AMOS: a demonstration. Int J Disabil Hum Dev. 2014;13. doi: 10.1515/ijdhd-2014-0305

[34] Chawla D, Joshi H. Scale Development and Validation for Measuring the Adoption of Mobile Banking Services. Glob Bus Rev. 2019;20:434–57. doi: 10.1177/0972150918825205

[35] Qi X, Pei Y, Wang K, Han S, Wu B. Social isolation, loneliness and accelerated tooth loss among Chinese older adults: A longitudinal study. Community Dent Oral Epidemiol. 2023;51:201–10. doi: 10.1111/cdoe.12727 35040179 PMC9288561

[36] Nordenram G, Davidson T, Gynther G, Helgesson G, Hultin M, Jemt T, et al. Qualitative studies of patients’ perceptions of loss of teeth, the edentulous state and prosthetic rehabilitation: A systematic review with meta-synthesis. Acta Odontol Scand. 2012:29;71. doi: 10.3109/00016357.2012.734421 23101439

[37] Abbas H, Aida J, Cooray U, Ikeda T, Koyama S, Kondo K, et al. Does remaining teeth and dental prosthesis associate with social isolation? A six-year longitudinal study from the Japan Gerontological Evaluation Study (JAGES). Community Dent Oral Epidemiol. 2023;51:345–54. doi: 10.1111/cdoe.12746 35352849

[38] Sikström S, Kelmendi B, Persson N. Assessment of depression and anxiety in young and old with a question-based computational language approach. Npj Ment Health Res. 2023:24;2:11. doi: 10.1038/s44184-023-00032-z PMC10955843 38609578

[39] Gerritsen AE, Allen PF, Witter DJ, Bronkhorst EM, Creugers NH. Tooth loss and oral health-related quality of life: a systematic review and meta-analysis. Health Qual Life Outcomes. 2010;8:126. doi: 10.1186/1477-7525-8-126 21050499 PMC2992503

[40] AL-Omiri MK, Karasneh JA, Lynch E, Lamey PJ, Clifford TJ. Impacts of missing upper anterior teeth on daily living. Int Dent J. 2009;59:127–32. doi: 10.1922/IDJ_1994ALOmiri06 19637520

[41] Besford JN, Sutton AF. Aesthetic possibilities in removable prosthodontics. Part 1: the aesthetic spectrum from perfect to personal. Br Dent J. 2018;224:15–9. doi: 10.1038/sj.bdj.2018.2 29326443

[42] Rana S, Kelleher M. The Dangers of Social Media and Young Dental Patients’ Body Image. Dent Update. 2018;45:902–10. 10.12968/denu.2018.45.10.902

[43] Campos LA, Campos JADB, Marôco J, Peltomäki T. Aesthetic dental treatment, orofacial appearance, and life satisfaction of Finnish and Brazilian adults. PLOS ONE. 2023;18:e0287235. doi: 10.1371/journal.pone.0287235 37384731 PMC10310051

[44] Andreas Zenthöfer A, Rammelsberg P, Cabrera T, Hassel A. Prosthetic rehabilitation of edentulism prevents malnutrition in nursing home residents. Int J Prosthodont. 2015;28:198–200. doi: 10.11607/ijp.4016 25822309

[45] Gotfredsen K, Walls AWG. What dentition assures oral function? Clin Oral Implants Res. 2007;18:34–45. doi: 10.1111/j.1600-0501.2007.01436.x 17594368

[46] Moynihan P, Varghese R. Impact of Wearing Dentures on Dietary Intake, Nutritional Status, and Eating: A Systematic Review. JDR Clin Transl Res. 2022; 7:334–51. doi: 10.1177/23800844211026608 34210202

[47] Salazar S, Hasegawa Y, Kikuchi S, Kaneda K, Yoneda H, Nokubi T, et al. The impact of a newly constructed removable denture on the objective and subjective masticatory function. J Prosthodont Res. 2021;65:346–52. doi: 10.2186/jpr.JPR_D_20_00045 33028800

[48] Shetty KB, Alshaqha EM, Koosa AB, Jambi SF, Jamal NO. Trends, Awareness, and Attitudes of Patients Towards Replacement of Missing Teeth in the Western Region of Saudi Arabia. J Clin Diagn Res. 2021;15:21–6. 10.7860/JCDR/2021/47926.14908

[49] Dosumu OO, Ogunrinde JT, Bamigboye SA. Knowledge of Consequences of missing teeth in patients attending Prosthetic Clinic in U.C.H. Ibadan. Ann Ib Postgrad Med. 2014;12:42–48. https://pubmed.ncbi.nlm.nih.gov/25332700/25332700 PMC4201933

[50] Tavakol M, Dennick R. Making sense of Cronbach’s alpha. Int J Med Educ. 2011;2:53–55. doi: 10.5116/ijme.4dfb.8dfd 28029643 PMC4205511

[51] International Test Commission. The ITC Guidelines for Translating and Adapting. 2017. Available from: https://www.intestcom.org/files/guideline_test_adaptation_2ed.pdf.

