# Supplementary material for: Psychometric properties of the Arabic version of the perceived prosthodontic treatment need scale: Exploratory and confirmatory factor analyses
Source: PLoS One. 2024 Feb 6;19(2):e0298145. doi: 10.1371/journal.pone.0298145 (PMC10846707; doi:10.1371/journal.pone.0298145)
Supplement: S1 File — (PDF) [file pone.0298145.s001.pdf]

Supporting information 1. The Arabic translation version of PPTN scale.

1. Have you ever avoided going out because of problems with your teeth or mouth?<br>هل سبق لك أن تجنبت الخروجَ من المنزلِ بسبب مشاكل في أسنانك أو فمك؟
2. Have you ever faced problems in social or leisure activities because of problem(s) with your teeth or mouth?<br>هل سبق لك أن وجهتَ صعوبة في المشاركة في الأنشطة الاجتماعية أو الترفيهية بسبب مشاكل في أسنانك أو فمك؟
3. Have you ever worried about what your partner, relatives, or friends think about your teeth?<br>هل سبق لك أن شعرتَ بالقلقِ من نظرة (زوجتك/زوجك) أو أقاربك أو أصدقائك لمظهر أسنانك؟
4. Have you ever felt upset when comparing teeth with others?<br>هل سبق لك أن شعرت بالانزعاج عند مقارنة أسنانك بالآخرين؟
5. Have you ever felt unhappy, sad, irritable, or depressed about the appearance of your teeth or mouth?<br>هل سبق لك أن شعرت بالحزن، أو الانفعال أو الاكتئاب بسبب مظهر أسنانك أو فمك؟
6. Have you ever felt shy or embarrassed because of any problem with your teeth or mouth?<br>هل سبق لك أن شعرت بالخجل أو الإحراج بسبب مشاكل في أسنانك أو فمك؟
7. Have you ever felt not confident because of your teeth or mouth?<br>هل سبق لك أن شعرت بعدم الثقة بسبب مظهر أسنانك أو فمك؟
8. Have you ever disliked seeing or avoided showing your teeth in the mirror or on photograph or video of yourself?<br>هل سبق لك أن كرهت أو تجنبت رؤية أسنانك أمام المرآة أو في الصور أو الفيديو الخاصة بك؟
9. Have you ever felt self-conscious about the appearance of your teeth?<br>هل سبق لك أن شعرت بالوعي الذاتي لمظهر أسنانك؟
10. Have you spent time observing or looking at your teeth or mouth?<br>هل سبق لك أن قضيتَ وقتاً في مراقبة أو النظرِ في أسنانك أو فمك؟
11. Have you ever disliked your teeth color?<br>هل سبق لك أن كرهت لون أسنانك؟
12. Have you ever disliked your teeth when you smile?<br>هل سبق لك أن كرهت مظهر أسنانك عندما تبتسم؟
13. Have you ever felt that your teeth are not attractive?<br>هل سبق لك أن شعرت أن أسنانك ليست جذابة؟
14. Have you ever had problem in chewing?<br>هل سبق لك أن واجهت مشكلة في المضغ؟
15. Have you ever avoided eating certain types of food due to your teeth or mouth?<br>هل سبق لك أن تجنبت تناول بعض الأطعمةِ بسبب مشكلة في أسنانك أو فمك؟
16. Have you ever felt discomfort due to food getting stuck in between your teeth?<br>هل سبق لك أن شعرت بعدم الارتياح بسبب تجمع الطعام بين أسنانك؟
17. Overall, how do you rate your need for dental treatments?<br>بشكل عام، كيف تقيم حاجتك لعلاج أسنانك؟
