# Supplementary material for: Psychometric properties of the Arabic version of the perceived prosthodontic treatment need scale: Exploratory and confirmatory factor analyses
Source: PLoS One. 2024 Feb 6;19(2):e0298145. doi: 10.1371/journal.pone.0298145 (PMC10846707; doi:10.1371/journal.pone.0298145)
Supplement: S2 File — (PDF) [file pone.0298145.s002.pdf]

The relevance ratings on the item scale by 6 experts

Item	t 1 MA (Pr	ert 2 AB (E	ert 3 RH (Re	4 HA (Ora	t 5 AQ (Pu	rt 6 AG (Oral S)	Experts in agreement	I-CVI	UA
Q1	1	1	1	1	1	1	6	**1**	1
Q2	1	1	1	1	1	1	6	**1**	1
Q3	1	1	1	1	0	1	5	**0.83**	0
Q4	0	1	1	1	1	1	5	**0.83**	0
Q5	1	1	1	1	1	1	6	**1**	1
Q6	1	1	1	1	1	1	6	**1**	1
Q7	1	1	1	1	1	1	6	**1**	1
Q8	1	1	1	1	1	1	6	**1**	1
Q9	1	1	1	1	1	1	6	**1**	1
Q10	0	1	1	1	1	1	5	**0.83**	0
Q11	1	1	1	1	1	1	6	**1**	1
Q12	1	1	1	1	1	1	6	**1**	1
Q13	1	1	1	1	1	1	6	**1**	1
Q14	1	1	1	1	1	1	6	**1**	1
Q15	1	1	1	1	1	1	6	**1**	1
Q16	1	1	0	1	1	1	5	**0.83**	0
							S-CVI/Ave	0.96	
Proportion relevance	0.87	1	0.94	1	0.94	1	S-CVI/UA		0.75

The average of proportion relevance scores across all experts	0.96

›n, we can conclude that I-CVI, and S-CVI/Ave meet satisfactory level, and thus the scale of questionnaire has achieved satisfac
